# Are Patient and Carer Experiences Mirrored in the Practice Reviews of Self-management Support (Prisms) Provider Taxonomy?

**DOI:** 10.5334/ijic.2483

**Published:** 2017-08-27

**Authors:** Nicolette Sheridan, Timothy Kenealy, Kerry Kuluski, Ann McKillop, John Parsons, Cecilia Wong-Cornall

**Affiliations:** 1 University of Auckland, NZ; 2 University of Toronto, CA

**Keywords:** self-management support, patient experience, carer experience, chronic conditions, community-based primary health care, integrated care

## Abstract

**Introduction:**

Patient self-management support is central to care for long term conditions and for integrated care. Patients and their carers are the final arbiter of whether support for self-management has been effective. A new taxonomy lists 14 categories of provider activities that support patient self-management (Practical Reviews in Self-Management Support, PRISMS). We asked whether we could recognise these provider activities in narratives from patients and carers. We sought to extend the theoretical framework of the taxonomy to include the view from patient and carers.

**Methods:**

We interviewed 28 patients and family carers in a case study of primary health care in New Zealand in 2015 to determine which components of the taxonomy were visible. We drew on interviews with clinicians and organisation persons to explain case study context.

**Results:**

We found, within patient and carer data, evidence of all 14 components of provider self-management support. The overarching dimensions of the taxonomy helped reveal an intensity and consistency of provider behaviour that was not apparent considering the individual components.

**Conclusions:**

Patient and carer data mapped to provider activities. The taxonomy was not explicit on provider relationships and engagement with, or separate support needs of, patients and carers.

## Introduction

“In chronic illness, day-to-day care responsibilities fall most heavily on patients and their families” [1]. People living with long term conditions, and their carers, make decisions about the actions they take that effect their health (self-management). Supporting people to better manage their conditions can improve their physical and mental wellbeing and change how they use services. A key role of health providers is to support self-management through the provision of basic information, shared decision-making, strategies to develop skills, and emotional support.

The best evidence suggests that supporting self-management can meet patient needs and has been linked to improved clinical outcomes and decreased emergency service use [2]. Self-management has been widely promoted as both increasing effectiveness and reducing the cost of care for long term conditions. Self-management support is one of the six elements of the Chronic Care Model identified as essential to an integrated health system that encourages high quality chronic disease care [3]. We would argue that self-management support is the most critical element of this foundational model (and therefore to integrated care) in that it is the final common pathway by which providers and organisations influence health outcomes for individual patients and populations.

Pearce and colleagues [4] have recently argued that researching, measuring, managing and improving self-management support has been hindered by inconsistent and incomplete descriptions and classifications of components of self-management. They synthesised the literature extending previous classifications to produce a new 14-item taxonomy, accompanied by four ‘over-arching’ dimensions, see Table 1. This taxonomy is confined to the actions of health care providers, who provide ‘direct’ self-management support to patients and their carers, while excluding the influences of organisations and other contexts which provide ‘indirect’ support (for example, by improving computer systems).

**Table 1:** PRISMS taxonomy (from Pearce and colleagues, 2016).

Component name	Component description

Component 1. Information about the condition and/or its management	Providing people with LTCs with information and instruction about their LTC or about general aspects and principles of managing their LTC (physiology, medication, prognosis, emotional, psychosocial, etc.).
Component 2. Information about available resources	Providing people with LTCs with information (e.g. written, verbal, visual) on issues such as financial benefits, sources of social or peer support, charitable organizations.
Component 3. Provision of/agreement on specific clinical action plan and/or rescue medication	Written instructions prepared with or by a health-care professional to enable the person to stay in control of their condition, tailored to the person, LTC, and severity. Includes how to take medication, recognize symptoms of deterioration and what actions to take.
Component 4. Regular clinical review	A regular, scheduled review of the person, their condition and self-management, ­conducted by a health-care professional.
Component 5. Monitoring of condition with feedback	Monitoring symptoms, behaviours or objective measures related to LTC. Can be done by the person with a LTC or by others but the results must be fed back to the patient. Interpretation, decision and/or action is undertaken by the patient, but may be supported by a professional. Professionals may support self-management by reviewing monitored data and providing feedback to the patient.
Component 6. Practical support with adherence (medication or behavioural)	Provision of practical help to improve a person’s adherence to medication or behaviour change activities.
Component 7. Provision of equipment	Provision of equipment to enable, assist or promote self-monitoring and/or self-management of the LTC.
Component 8. Provision of easy access to advice or support when needed	People with LTCs are provided with flexible access to and timely advice from health services in the event of an urgent or non-urgent question or concern arising.
Component 9. Training/rehearsal to communicate with health care professionals	Teaching people with LTCs to develop communication skills/techniques to improve relationships, better communicate needs, and enhance shared decision making with health-care professionals. Also supporting/mentoring people with LTCs to practise the skills they have been taught.
Component 10. Training/rehearsal for everyday activities	Teaching people with LTCs to develop skills that support everyday activities and/or supporting people with LTCs to practise the skills they have been taught.
Component 11. Training/rehearsal for practical self-management activities	Teaching people with LTCs to develop specific practical skills that will enable them to manage their LTC, and/or supporting people with LTCs to practise the skills they have been taught.
Component 12. Training/rehearsal for psychological strategies	Teaching people with LTCs skills in using psychological strategies to help them better manage the consequences of an LTC and/or supporting them to practice the skills they have been taught. May include: problem-solving strategies, relaxation techniques, re-framing, distraction, cognitive restructuring, goal setting and action planning (prompts detailed planning of performance of the behaviour/outcome of the behaviour, NB this does not have to be health behaviour focussed).
Component 13. Social support	Facilitation of social support, where a person feels cared for and supported by others in a social network. May include befriending, peer support, peer mentoring and group socializing.
Component 14. Lifestyle advice and support	Provision of advice and support around health and lifestyle. Relates to practical advice and support in relation to handling life stressors, NOT psychological elements that relate to handling life stressors (see A12 for training/rehearsal in psychological strategies). May include general lifestyle advice and support concerning diet, physical activity, smoking cessation, and alcohol intake.

Self-management is something patients with long term conditions, and their families, do all the time. Self-management support is something that clinicians and others do only occasionally for any one patient. The patient must be the final arbiter of whether self-management support has been experienced and whether it has been effective. The literature from which the taxonomy was drawn was provider-centred rather than patient-centred, so that the language and concepts might not be readily recognised by patients.

We asked whether we could recognise a clear reflection of provider taxonomy activities in narratives from patients and carers. We had the opportunity to investigate this question with patient and carer data from a case study of a primary health care organisation that the clinician interviewers considered to be high-performing.

## Methods

### Setting

Data came from a case study of community-based primary health care in New Zealand in 2015. This was one of a series of case studies in New Zealand and Canada investigating implementation of innovative community-based primary health care. The case study organisation was established as a not-for-profit community trust almost two decades ago. It delivers primary care and public health services to an impoverished urban and rural region in the north of New Zealand comprising more than 20,000 people, of whom about 5,000 are indigenous Māori, and who are the focus of this service. Clinical staff included doctors, nurses, allied health professionals and community health workers. The senior doctors and nurses have post-graduate training. The organisation keeps good data that has earned them the respect of local secondary care services.

### Participants

Consumer participants were patients, or family carers, who were selected for variation by ethnicity (Māori or Other – mostly New Zealand European) and gender. Some carers cared for patients in this study, others cared for patients who were not in the study but met the same eligibility criteria. Patient participants were 50 years of age or older and had two or more long term conditions. Māori over the age of 50 have poorer health outcomes and a higher burden of multi-morbidity than non-Māori of the same age, which is often compounded by relative poverty [5]. We also interviewed providers, managers and policy-makers within the same case study.

### Data collection

Patient and carer interviews were conducted using a semi-structured questionnaire, mostly asking for a semi-quantitative response followed by an explanation which the interviewer was free to explore. Questions were derived from a number of validated questionnaires: Activities of Daily Living Scale [6]; Patient Assessment of Chronic Illness Care (PACIC) including five addition questions related to behaviour change [7–8]; Carer Reaction Assessment [9]; Cultural Justification for Care-giving Scale [10], and Hua Oranga (measuring Māori mental health outcomes) [11]. Additional supplementary questions related to personal characteristics, health and social history, relationship with health providers, material standard of living [12] and were derived from consensus discussions by researchers. Thirty three people volunteered in response to posters and signs on receptionists, desks in primary care practices. Five were too sick, not available or did not meet our inclusion criteria. Participants were interviewed at a place of their choice, commonly either a healthcare facility or in their own home. Participants could invite a family member/support person to attend the interview. All participants gave written consent to their interview being recorded. They could stop at any time or skip questions. Four researchers, one of Māori descent, conducted the interviews which lasted from 45 to 90 minutes. All participants chose to be interviewed in English. Digital audio file names were coded to ensure anonymity after interview. Interviews were transcribed verbatim omitting personal names. Ethics approval was given by the University of Auckland Human Participants Ethics Committee, reference 013071.

### Analysis

Quotes were selected from patient and carer interviews where we judged (by author consensus) they reported or commented on provider activity. Our goal was to test whether each component was reflected in patient and carer data. We selected quotes which were representative of many more. Beyond that, no attempt was made to systematically analyse our source data, to quantify quotes that fitted categories or to prioritise components. Additional data were drawn from interviews with providers and other organisation staff only to ensure we correctly understood some patient data, such as roles of providers mentioned by name. No attempt was made to formally codify these data.

Quotes have been purposively selected to illustrate the range of provider activities considered by patients and carers to support self-management, and to show that these perceptions came from the full range of participants: patients and carers; by age, gender and ethnicity; and by provider type – doctor, nurse, nurse practitioner and community health worker.

## Results

We interviewed 15 patients and 13 family carers, described further in Table 2. We have listed the 14 taxonomy components with accompanying quotes from patients or carers that relate to provider activities in each component. More quotes are cited for components where there are a wide range of examples. The quotes chosen reflect the diversity of patients and carers across genders, age groups and generations. Explanatory data came from interviews with 7 clinicians, 3 managers and 5 policy persons.

**Table 2:** Describing patients and carers. Results are all counts.

Role	Māori	Female	Age	Generations in household

Patient (n = 15)	14	6	50–64 6 65–75 5 ≥75 4	1 generation 9 2 generations 2 3 generations 4
Family Carer (n = 13)	8	11	35–49 3 50–64 8 65–75 1 ≥75 1	1 generation 4 2 generations 6 3 generations 2 4 generations 1

The original taxonomy called for an assessment of each component against four overarching dimensions – mode of delivery; the personnel delivering or facilitating the support; the targeting of the intervention; and the intensity, frequency and duration of the intervention. Because provider performance seemed to fall into similar patterns across many taxonomy components, we have instead provided an overall summary of our findings against each of these dimensions, together with further commentary in the Discussion.

### Component 1. Information about condition and/or its management

“He told me I had to start taking blood pressure tablets, I was not at all impressed. And I explained to him that I wasn’t comfortable about taking medication, and he went through the process of talking through it. Then he gave me a script and he said it was my choice to consider. And he said, ‘You can keep this. You can take it to the pharmacy and they can hold it for you. And, if you don’t use it, that’s okay.’ It took me several months before I was even able to think about swallowing that pill. But in the end I went after having a few more discussions with him.… Yes, there is that ‘confident’ word – I felt confident enough to choose to take it, because of the options that he had explained carefully to me” (Female patient, 50–64 years).

“[Nurse Practitioner] recommended I do a shopping… a supermarket visit with a dietician…. I am taking more care to look at what’s on the back of the tin I pick up, so I’m looking for low salts, low sugars… even in the drinks… fizzies and all that. So, I guess I have taken on some of the commitment” (Female patient, 50–64 years).

### Component 2. Information about available resources

“I couldn’t afford that, she’ll give me the pills to start me off, and then she’ll make sure I get two or three of my pills on the one script for only $5. Or she sends me to a chemist down in [location], because I said to her, ‘Oh, I can’t afford that,’ and she goes, ‘Well, you really need it.’ So she puts a number on my prescription and tells me go down to [location] in town, and I get it for free” (Female patient, 50–64 years). Comment: The Nurse Practitioner and organisation understood the severe hardship some patients and their families experienced, and in an effort to support adherence to medicines, worked with the local pharmacy to subsidise the cost to the patient.

“Yeah, they give you an accommodation supplement, I get $80, and this place is $370 a week, so it’s not much, but it does help, anything helps…. I only go there as a last resort… you have your emotions and everything at the door when you walk in, because you feel so terrible that you’ve got to go there and ask for this and ask for that” (Male patient, 50–64 years). Comment: Community health workers often went with patients to practically assist them with filling in forms at welfare and social agencies, but perhaps more importantly, to provide emotional support.

### Component 3. Provision of/agreement on specific clinical action plan and/or rescue medication

“Yep, we’ve got a plan, it’s called a disaster plan… we update it every so many months” (Male patient, 50–64 years). Comment: This referred to a written acute treatment plan.

“if it wasn’t working here, they wouldn’t have to ask me about it. I’d be telling them, ‘Look, something is wrong here. Either your plan for us is wrong, or we are mismanaging the plan. Let’s go through this agreement again and see where we are not cutting it’” (Female carer, 65–74 years). Comment: Some carers, and some patients, had high expectations of health providers as the architects of a “good plan” and that the responsibility for management was not solely theirs.

### Component 4. Regular clinical review

“I’m due for my three-month check, nothing usually at the moment crops up with me from three months to three months…” (Female patient, 65–74 years). Comment: This patient refers to her CarePlus check, which is designed to improve chronic care management. In the national CarePlus programme, a patient has an initial comprehensive assessment where their health needs are identified, a care plan is developed, and there are regular follow-ups.

### Component 5. Monitoring of condition with feedback

“I just couldn’t breathe, and I’d been sick for about three weeks… I came in, she came out and gave me a pump and a spacer, showed me how to use it, gave me some antibiotics and told me to come back the next day, if I get any worse” (Female patient, 50–64 years).

“So they’re monitoring my high blood pressure. And the medication they’ve given me, they’ve explained to me what it is for. And because I understand what it is for now, I take them. Whereas before, oh no, didn’t know, so out the window it went” (Female patient, 50–64 years).

### Component 6. Practical support with adherence (medication or behavioural)

“Well she was taking wrong medication, and yeah, making herself sick but she didn’t mean to. But you know she just couldn’t see, so I had a talk to the doctor and then we went to have a talk to the chemist and the chemist suggested that we blister-pack them” (Male carer, 50–64 years). Comment: The carer is the husband of a woman who had detached retinas, renal failure and was on renal dialysis.

“I got her [Nurse Practitioner] phone number in my phone and that. Finding anything I can just ring her and, she will, I don’t even need to see her she will just, I ask her what I need, she will make up the prescription and fax it through to the chemist and all I’ve got to do when I have time is go to the chemist and pick it up. So yeah, she is an amazing lady” (Male carer, 50–64 years). Comment: Because the Nurse Practitioner was fully aware that the patient lived rurally and that it was often difficult to collect a repeat prescription she made it as easy as possible for the patient to collect and adhere to her medication regime.

### Component 7. Provision of equipment

“they sent me home, they said I wasn’t allowed to work, walk or anything, and we didn’t have the money for the crutches. It was 20 bucks, and I know that doesn’t sound much but…. if it’s not after our pay day, we don’t have $10. So [Nurse Practitioner] organised that, plus the wheelchair and everything. I know, isn’t that fabulous. It was quite stressful… me with two broken feet [laughs]” (Female carer, 50–64 years). Comment: The Nurse Practitioner phoned the carer after her discharge from hospital and then home visited, following-up with crutches etc. and also seeing the index patient (husband).

### Component 8. Provision of easy access to advice or support when needed

“[Nurse Practitioner] told me to come back the next day, if I get any worse just to come back and see her. And there was no clinic. And she still saw me” (Female patient, 50–64 years). Comment: This patient valued the relationship she had with the Nurse Practitioner and described her relief and confidence in being able to rely on her.

“I get 8 hours home care. And I have 12 hours personal care if I need it. But I don’t need any help with showering or anything like that but sometimes if I’ve got an appointment at the hospital, on my days off, she [community health worker] would ring me up and drive me because I am shattered you know” (Male carer, 50–64 years).

### Component 9. Training/rehearsal to communicate with health care professionals

“I realized the importance of anti-inflammatory and [Nurse Practitioner] was very, very patient with me, explaining why I needed to take an anti-inflammatory. And, my daughter I must say, would constantly say to me, “Mum, you need take an anti-inflammatory.” And, I say, “No, I’m not taking one of those!” When [Nurse Practitioner] explained it, I just realized…” (Female patient, 50–64 years).

“my wife got diagnosed with diabetes… I was actually more upset than her… I actually thought diabetes meant [pause] you know you are going to die. I didn’t know anything about it… then [Doctor] talked us through and he explained everything to us and the nurses helped us out.” (Male carer, 50–64 years).

“We’ve had a thorough talk, that’s the point, though, we’ve had a thorough talk, so the choices I’m making now are my choices based on the information that I’ve been given. It’s up to me to make a better choice” (Female patient, 50–64 years).

### Component 10. Training/rehearsal for everyday activities

“[Nurse] does, she thinks it’s important to exercise and she has impressed that on me. And, I do think I do quite a bit of exercise. If I wasn’t injured, I would be involved in exercise every day of the week. I mean, on Monday, with my yoga and tai chi. Wednesday was my chi gong and asthma group. On Thursday, Sit Fit … I want so much to get back to my routines” (Female patient, 50–64 years).

“Yes. She told me to walk up my hill [laughter]. “Oh, it’s only half an hour, just walk up, around and around.” And it did make a big difference. And it made an even bigger difference when I stopped smoking. Because now it’s easy for me to walk up the hill and back down again” (Female patient, 50–64 years).

### Component 11. Training/rehearsal for practical self-management activities

“Yeah, my level of sugar, glucose. Because I’ve got one of those [glucose monitor] where you prick it, and I’m always round about 7 points, and the highest is about 8.2. I do that every day, and I go for my glucose… it’s not too bad, you know” (Male patient, 65–74 years). Comment: This man was taught to use his glucose monitor when he first enrolled with the case study organisation, despite having diabetes long before joining.

“But, I mean, prior to having that stroke, the symptoms, I didn’t know what the symptoms… what to look for, but now I certainly know” (Female carer, 35–49 years). Comment: This carer was shown how to help the patient with activities of daily living.

### Component 12. Training/rehearsal for psychological strategies

“Yes, she has learnt to manage that a lot better, through going to [Doctor]. Because she let it get her down, she will be down in the dumps for two days. Whereas now she has learnt signs and you know, the early stages of it so she can sort of bring herself out before she gets in there” (Male carer, 50–64 years).

“And so, yeah, it kind of came to a… a head, I guess, with Dad, that Mum and Dad sought some counselling to help with some issues, and that helped. But Dad being old school…; it helped a little, and then he forgot. Yep. Yep. So, it’s a constant journey for me…” (Female carer, 35–49 years).

### Component 13. Social support

“he likes him because [husbands name] has finally got it in his head that he’s trying to help him. Yeah, he trusts him. And [my husband] has got a big trust issue. There’s not many people he can trust, whatsoever. Because he’s telling [husband] how it is. He’s not pushing. You know, ‘You don’t want to come down to the doctor, I’ll come to you.’ He’s not pushing him. He’s working with him. That’s what it is. He’s working with him. That’s what it is” (Female carer, 50–64 years). Comment: This man had become so mistrustful of social services that the doctor became the first level of social support. He was later able to connect to mainstream services.

### Component 14. Lifestyle advice and support

“But then after the physio treatment, she felt she needed something more; she really wanted exercise, she wanted to get on a treadmill and start walking and doing this and that and the other. So, I had a talk to [organisation] again and they told me about a green prescription that we could get, and because I’m diabetic as well, that both Mum and I could get one. And I thought, “Well, that’s brilliant, because then I can go along to the gym and so can Mum, and we can both exercise and I can make sure she is okay along with the staff at the gym” (Female carer, 35–49 years). Comment: A Green Prescription is written advice to a patient or their family by a health professional to support them to become more physically active as part of a total health plan.

### Overarching dimension – modes of delivery (e.g. face-to-face, remote, telehealthcare, web-based)

Care was based on relationships between providers and both patients and carers. Once established, care may be extended by phone, email and messaging via family members - not so much to save provider time as to save patient and carer time and cost, particularly for those living rurally. Providers, patients and carers were equally clear that a relationship required providers to know and care about the personal, economic and socio-cultural circumstances of patients and carers. The Māori cultural context of this organisation, and of the community it serves, places specific emphasis on the value of face-to-face communication, and has a specific term for it (kanohi ki te kanohi).

### Overarching dimension – personnel delivering the support (e.g. health-care professionals, lay educators)

Care was delivered by a range of health care professionals employed directly by the organisation (general medical practitioners, a Nurse Practitioner, practice nurses, a community health worker, a podiatrist and a physiotherapist), and others either self-employed or employees of different organisations who worked in close coordination with the case study organisation (pharmacists, allied health workers, social workers, and hospital doctors and nurses). Many community support services were based at local marae (cultural meeting-place). These included exercise classes, weaving activities, visiting health providers and social services – all contributing to a sense of community and support.

### Overarching dimension – targeting (e.g. individual or groups, generic or condition-specific, cultural groups)

This organisation provided care that was centred on individuals within their whānau (extended family). Weekly multidisciplinary meetings reviewed clinical care of current patients. Monthly multidisciplinary meetings used data to pro-actively monitor and respond to needs of the organisation’s population. The organisation was a creation of, and responsible to, a specific Māori sub-tribe. Māori values, and a response to the endemic poverty of the population served, permeated the organisation and were routinely reflected in behaviour of health care providers and their clients. For example, the cost of petrol and /or not having a car was one of the commonest barriers to patients and carers attending urban clinics or picking up prescriptions from the pharmacy. In response, providers hold clinics in rural regions and visit some patients at home.

### Overarching dimension – intensity, frequency and duration of the intervention (not the individual components)

Care was perceived by patients and carers to be personalised and highly satisfactory, i.e. the right intensity, duration and frequency for them. This was supported by our observation and interviews with providers and others. We saw care that was highly competent and delivered flexibly to meet the clinical and personal needs of individual patients and carers. We saw appointment times offered, a flexible service response to people arriving without appointments, clinical priority given to people with urgent needs, care given in person or delegated, care delivered in a clinic or a home, with systematic follow up. Care continued when the patient was not present, with systematic multidisciplinary discussion of individuals and the population served.

## Discussion

Within patient and carer data from a case study of one primary health care provider organisation, we found evidence fitting all 14 components in a newly published taxonomy of provider self-management support activities. This taxonomy was intended to categorise all the modes of self-management support reported in the literature. There was no expectation that every provider meet every taxonomy component. Patient and carers in this case study describe high performance by the providers, reflected in activities that fit taxonomy components, and mirrored in the overarching dimensions of the taxonomy.

Providers will have their own views on whether they have provided, intended or attempted to provide self-management support. Only patients can attest to outcomes that are experienced by them and are important to them. Starting with patient and carer data has enabled us to see provider activities through a patient lens and a carer lens. We would argue that a relationship of trust between provider and patient and carer – engagement – must be present for any self-management support to be effective [13], and we would argue that the only pathway between cause (provider and organisation activities) and effect (on patients and carers) is via their choices and acts of self-management.

Patients and carers do not necessarily talk about provider activities – they talk of their experiences and outcomes and the researcher must infer the provider activities. It became apparent that, although we could readily identify “basic” provider activity in each component, such as providing information by handing a leaflet to a patient, we repeatedly heard stories of activities that went well beyond the “basic”, such that the basic was not even named. We needed to assume the basic activity had occurred or been superseded. For example, Component 1 calls for provision of “information and instruction”. We heard stories of sophisticated information giving and receiving that responded to the cultural and social context of the patient, providers partnering in a way that led to shared decision making, with full respect for patient dignity and choice.

Component 2 provides a similar example. It asks whether providers give information about resources to support patients. Our patients and carers report active advocacy strengthened by local knowledge and an understanding of their life circumstances, in particular, the distress related to financial hardship. Similarly, Component 3 asks about written instructions to enable the patient to control their condition. One carer quote indicates self-management support that goes well beyond treating the patient and carer as passive recipients of written instructions. It revealed a “power-with” relationship [14] guided by the broad goal of a dying family member whose most important desire is to spend time with grandchildren.

Quotes selected to illustrate Components 5 (monitoring and feedback) and 6 (practical help) show providers responding to cultural context, costs and patient adherence, with care integration between providers and appropriate follow up. For Component 9, rather than teaching patients to communicate with some other health care professionals our quotes show providers actively role-modelling skilful engagement [13]. Patients and carers repeatedly acknowledged receiving emotional support that was immensely valuable.

The full scale of the providers’ actions emerges only when considering the overarching dimensions of the taxonomy. The providers offer personal engagement, sharing of power and a long-term relationship. Together these translate into provider-patient-carer concordance, that was often identified as a new experience by these patients and carers, and which resulted in practical behaviour choices and changes summarised by the person who said (of their medication) “now I will take it”.

If this work is confirmed with other patients and carers, we think we offer a substantive extension to both the theory captured in the taxonomy and its practical uses. A criteria-set by which to assess provider self-management from the lens of provider, patient and carer could be valuable to all who plan, implement, measure and monitor self-management.

Limitations of the taxonomy, acknowledged by the authors, include their explicit exclusion of “indirect” support for self-management. Further work is needed to define the support that might come from organisations, funders and policy-makers, and, we would add, from peers and community. They also note that their taxonomy has nothing to say about the relative importance of different components of self-management support, and they have nothing to say about effectiveness, cost-effectiveness, relative effectiveness or synergy between components of the provider taxonomy.

Limitations of this study include sourcing patient and carer data from only one case study, albeit of a high-performing organisation. Future research, based on a larger dataset of patients and carers, might search for provider activities that may be missing from or under-developed in this taxonomy. For example, the taxonomy makes little distinction between supporting patients and supporting carers. Our interviews showed very different needs and experiences of patients and carers, and different supports offered by providers. It was also clear that carers played multiple important roles in supporting patient self-management.

## Conclusion

The PRISMS taxonomy achieves what it intends, and acknowledges important considerations that lie outside its self-defined scope. It is not explicit, however, on the most important consideration of patient self-­management ­support, a relationship and engagement with the patient. Further work is needed to elaborate the differences between self-management support for patients and support for carers. By bringing a patient and carer dimension to the taxonomy we offer the beginning of a substantive extension of the theory and practical use for the taxonomy.
